# Risk and resilience profiles and their transition pathways in the ABCD Study – CORRIGENDUM

**DOI:** 10.1017/S0954579425000094

**Published:** 2025-03-03

**Authors:** Ruiyu Yang, Sabrena Tuy, Lea Rose Dougherty, Jillian Lee Wiggins

**Keywords:** Developmental psychopathology, risk and resilience, person-centered approach, corrigendum

This article was originally published with an error in the acknowledgements. This has now been corrected and this corrigendum published. The correct acknowledgements are below:
